# Probing Functional Heteromeric Chemokine Protein–Protein Interactions through Conformation‐Assisted Oxime Ligation

**DOI:** 10.1002/anie.201607036

**Published:** 2016-10-27

**Authors:** Stijn M. Agten, Rory R. Koenen, Hans Ippel, Veit Eckardt, Philipp von Hundelshausen, Kevin H. Mayo, Christian Weber, Tilman M. Hackeng

**Affiliations:** ^1^ Department of BiochemistryUniversity of MaastrichtCardiovascular Research Institute Maastricht (CARIM)Universiteitssingel 506229 ERMaastrichtThe Netherlands; ^2^ Institut für Prophylaxe und Epidemiologie der KreislaufkrankheitenLudwig-Maximilians-Universität (LMU) MünchenPettenkoferstraße 8a und 980336MünchenGermany; ^3^ Department of BiochemistryMolecular Biology, and BiophysicsUniversity of Minnesota7-142 MCB420 Washington Ave SEMinneapolisMN55455USA

**Keywords:** atherosclerosis, chemokines, oximes, protein–protein interactions, solid-phase synthesis

## Abstract

Protein–protein interactions (PPIs) govern most processes in living cells. Current drug development strategies are aimed at disrupting or stabilizing PPIs, which require a thorough understanding of PPI mechanisms. Examples of such PPIs are heteromeric chemokine interactions that are potentially involved in pathological disorders such as cancer, atherosclerosis, and HIV. It remains unclear whether this functional modulation is mediated by heterodimer formation or by the additive effects of mixed chemokines on their respective receptors. To address this issue, we report the synthesis of a covalent RANTES‐PF4 heterodimer (termed OPRAH) by total chemical synthesis and oxime ligation, with an acceleration of the final ligation step driven by PPIs between RANTES and PF4. Compared to mixed separate chemokines, OPRAH exhibited increased biological activity, thus providing evidence that physical formation of the heterodimer indeed mediates enhanced function.

Chemokines are small chemotactic cytokines that mediate leukocyte trafficking, which makes them interesting pharmaceutical targets to antagonize inflammatory diseases such as rheumatoid arthritis, Crohn's disease, and atherosclerosis.^1^ By binding to their cognate G protein‐coupled receptors, they can rapidly trigger leukocyte chemotaxis and adhesion, and thus regulate a variety of cellular processes for example, angiogenesis, embryonic development, and cell homeostasis.^2^

The structure of chemokine monomers is characterized by a disordered N‐terminus and three‐stranded antiparallel β‐sheets onto which a C‐terminal α‐helix is folded. Based on the spacing between conserved N‐terminal cysteine residues, chemokines are divided into four groups, with the major ones being the CC‐ and CXC‐types. Although small (<10 kDa), most chemokines have the propensity to form homodimers or higher‐order multimeric structures (>200 kDa).^3^ While the monomeric structures of all chemokines are highly conserved, there are notable differences between their (oligomeric) quaternary structures. For example, CC‐type chemokine dimers, formed by interactions between their N‐termini, are elongated and dumbbell shaped, whereas CXC‐type chemokine dimers are formed by interactions between their central β‐sheets, which makes them more globular in structure.

The chemokines RANTES (CCL5) and platelet factor 4 (PF4, CXCL4) are stored in the α‐granules of platelets. While RANTES is a potent attractant of mononuclear cells, PF4 lacks classical chemo‐attractant functions.^4^ However, the presence of PF4 can synergistically enhance RANTES‐mediated cell‐recruitment mediated by heterophilic interactions between chemokines.^4^ In fact, pharmacologic inhibition of RANTES and PF4 heterodimer formation inhibits monocyte recruitment and attenuates progression of atherosclerosis, lung injury, and aneurysm formation in mice.^5^ Heterophilic chemokine interactions have also been reported for CXCL4 and CXCL8,^6^ CCL19 and CCL22,^7^ CCL2 and CCL8,^8^ as well as other chemokines.^9^ These studies support the proposal that heterophilic interactions between different chemokines are relevant for many physiological and pathological processes.

Although current evidence suggests that physical formation of chemokine heterodimers mediates their observed functional modulation, the possibility remains that this could simply be due to combined receptor stimulation by the respective chemokines. Therefore, the aim of the present study is to generate an obligate RANTES‐PF4 heterodimer by total chemical synthesis to address this crucial mechanistic question.

Based on the observation that RANTES and PF4 interact through their N‐termini as a CC‐type dimer,5a we used this model as a starting point to assess optimal coupling strategies for the protein pair (Figure 1). Because oxime ligation can be performed with folded proteins in aqueous buffer at neutral pH, we chose it to covalently link the two proteins. Oxime ligation requires the incorporation of a ketone in one protein and an aminooxy moiety in the other. Owing to limitations in the ketone functionality of the widely‐used levulinic acid, we used 5‐ketohexanoic acid because of its high reactivity and conformational flexibility.^10^

**Figure 1:**
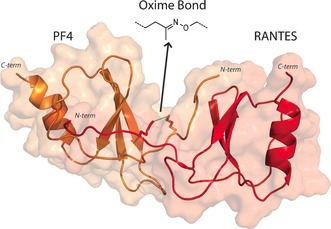
Schematic of a covalently linked dimer of PF4 (left) and RANTES (right). The termini of the two chemokines are labeled. The oxime connection between the two proteins is shown.

The proposed heterodimer consists of a RANTES monomer (68 aa) that is covalently linked to a PF4 monomer (76 aa) (Figure 2). The chemokines were each assembled by two subsequent native chemical ligations (NCL),^11^ followed by oxidative folding, and joined by oxime ligation. Positions suitable for covalent linkage of the two proteins were based on the proposed heterodimer structure observed by NMR spectroscopy.5a Furthermore, the N‐terminus of RANTES was required to remain unrestricted to allow interactions with its receptor (CCR5).^12^ Additionally, amino acids crucial for RANTES‐PF4 interaction were left untouched, leaving Thr7 in RANTES and Leu8 in PF4 as a site for crosslinking (Figure 1). Therefore, Thr7 and Leu8 were replaced by orthogonal protected lysines to provide a handle for introducing the ketone or aminooxy moiety. All reactions were monitored by HPLC‐MS. After oxidative folding of the chemokines to obtain modified RANTES (**16**) and PF4 (**8**), an oxime linkage between the two proteins was obtained by reacting equimolar quantities of the two modified chemokines (200 μm) in aqueous buffer at pH 4.5. This led to the desired obligate PF4‐RANTES heterodimer (OPRAH, **17**) with a 60 % yield after 12 h (Figure 2; Supporting Information, Figures S1 and S2). A functional negative control (nOPRAH) was established by linking Ser1 of RANTES to Glu1 of PF4 through an identical oxime linker by which the N‐terminal interaction of RANTES with its receptor CCR5 was blocked.

**Figure 2:**
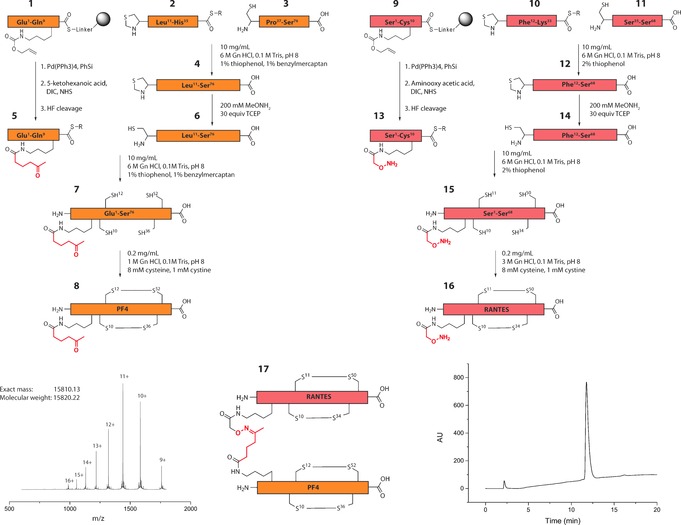
Schematic of the obligate PF4‐RANTES heterodimer (OPRAH) synthesis. Insets show the mass spectrum of **17** (left) and the UV‐detected LC chromatogram of **17** (right). Both C‐terminal peptides (**3** and **11**) were synthesized using standard Boc solid‐phase peptide synthesis protocols.^13^ Sequential NCL required N‐terminal encrypted cysteines (thiaprolines) to be used in the middle fragments (**2** and **10**).^14^ Both N‐terminal fragments (**1** and **9**) contain a thioester for NCL and require an orthogonally protected (N*ϵ*‐Alloc) lysine for incorporation of the ketone or aminooxy functional group. Deprotection of the Alloc group was performed on resin using Pd(PPh_3_)_4_, and the ketone (5‐ketohexanoic acid) and aminooxy (Boc‐(aminooxy)acetic acid) moieties were introduced to obtain **5** and **13**, respectively. After synthesis and purification, all peptide fragments were ligated sequentially using 1–2 % thiophenol and benzylmercaptan as catalysts.

In an effort to improve the reaction rate, we observed only minor enhancement by addition of aniline as a catalyst.^15^ To rule out that homomeric interactions between folded chemokines would impair heteromeric oxime formation, we performed oxime ligation in the presence of a denaturing agent (6 m Gn⋅HCl). Surprisingly, instead of accelerating heteromeric oxime formation, the presence of Gn⋅HCl slowed down oxime ligation 5‐fold, whereas it only had a minor effect on the reaction rate in a model system (Figure 3; Supporting Information, Figure S3). This observation indicated that the rate of formation of the oxime bond between RANTES and PF4 was accelerated by heterophilic chemokine interactions, which bring the two reactive species into close proximity (Figure 3). In retrospect, the proposed conformation‐assisted ligation was supported by the lower‐than‐expected catalytic effect of aniline. Eventually, using slow freezing catalysis,^16^ we were able to achieve complete oxime reaction within 2 h.

**Figure 3:**
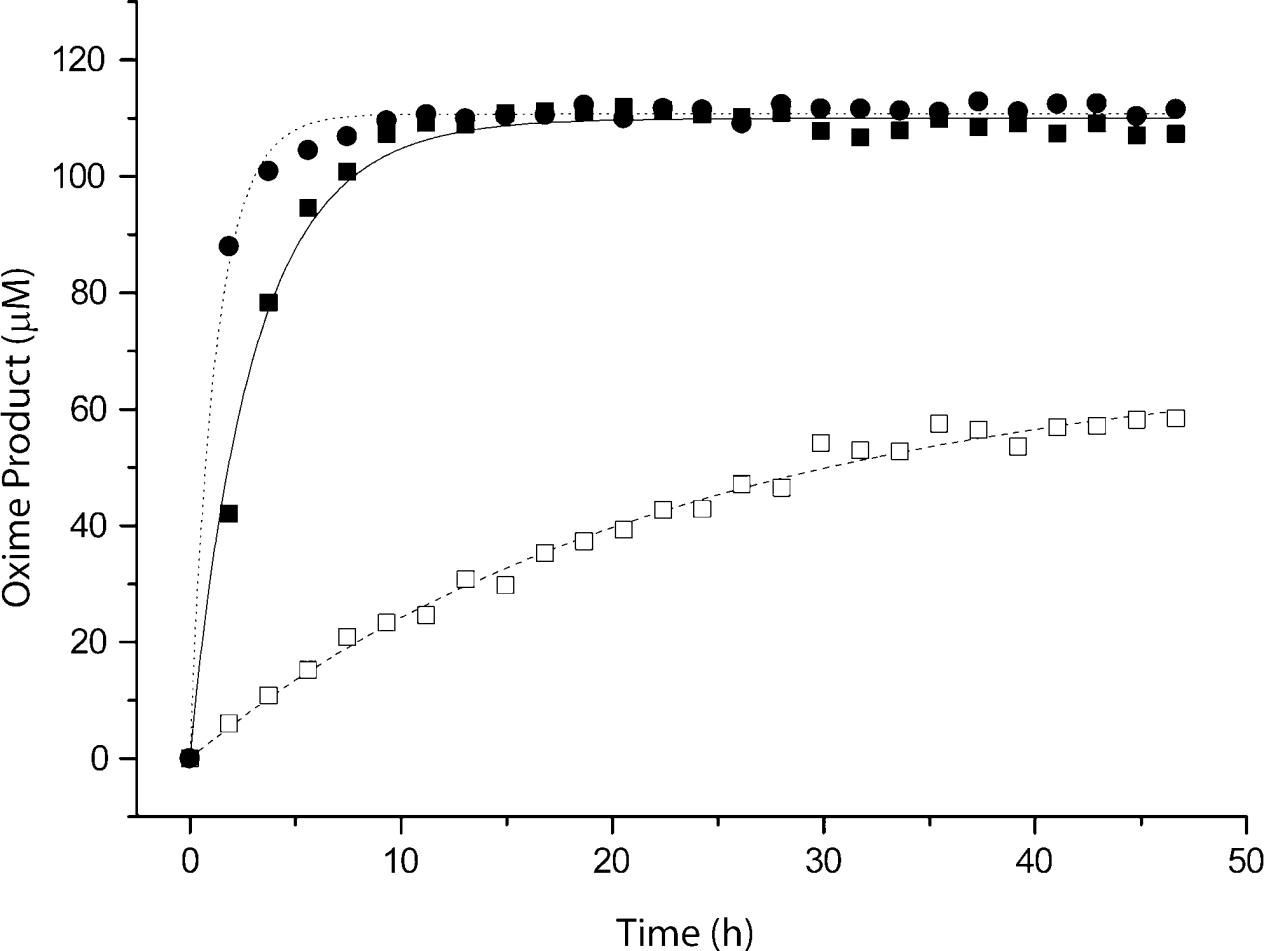
Oxime formation between **8** and **16** monitored over time in 0.1 m NaOAc without additions (▪) and with the addition of 6 m Gn⋅HCl (□) or 100 mm aniline (•).

To assess the folded states of modified PF4 and RANTES, as well as OPRAH, we used NMR spectroscopy. NMR spectra show that modified PF4 and RANTES are well folded. However, modified PF4 could still form tetramers like wild type PF4, whereas modified RANTES with its N‐terminally positioned lysyl‐linker showed markedly attenuated dimer formation. Compared with the NMR spectra of the non‐ligated proteins, that of OPRAH appears to show two correctly folded domains of the respective chemokines (Figure 4, Supporting Information, Figure S4). The resolved Trp57 H*ϵ* resonance in the spectrum of **16** is a good indicator of the folding preference of the RANTES subunit in OPRAH. In non‐ligated RANTES, Trp57 H*ϵ* resonances are observed for monomer (m) and homodimer (d) states (inset, Figure 4). Upon covalent dimerization of **16** and **8** to form OPRAH (**17**), the RANTES homodimer d state peak decreases in intensity, whereas the m peak increases and has a slight chemical shift, which is consistent with a change in chemical environment likely due to formation of the linked heterodimer. The remaining RANTES d peak intensity in **17** may arise from some homodimerization of two RANTES domains in OPRAH.

**Figure 4:**
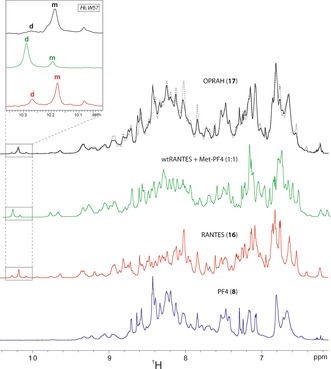
Comparison between 700 MHz 1D ^1^H NMR spectra (amide region) of PF4 (**8**), RANTES (**16**), OPRAH (**17**) and a 1:1 complex of wtRANTES and Met‐PF4, under similar conditions. Top spectrum: Overlay of experimental ^1^H NMR spectrum of OPRAH (black) and the sum of the spectra of free RANTES (**16**) and PF4 (**8**) (broken line) The agreement between experimental spectrum **17** and the summed spectra **8**+**16** shows that, globally, OPRAH consists of two natively folded PF4 and RANTES protein domains. Local differences are, however, apparent and these regions correspond to sharp amide peaks in the N‐terminus region (amino acids 2–7) of free PF4 that seem to broaden out significantly in OPRAH. Inset: Trp57 H*ϵ* signal is indicated for free RANTES (**16**), the 1:1 complex, and OPRAH (**17**).

We also followed real‐time oxime formation between **8** and **16** by NMR spectroscopy (Supporting Information, Figures S4–6). These NMR spectra show overall resonance broadening, which we attribute to transient formation of the OPRAH heterodimer. This is supported by our observations with the H*ϵ* resonances of Trp57, which is an indirect structural probe owing to its distance from the modified N‐terminus of **16**. Overall, we conclude that the general folds of the two protein domains, PF4 and RANTES, are maintained in OPRAH, and resonance broadening of the residues at the oxime ligation site suggests dynamic interactions between the two covalently‐linked domains.

We next investigated the biological activity of OPRAH in a monocyte arrest assay. A monolayer of endothelial cells was pre‐treated with chemokines or heterodimers, after which monocytes were passed over the surface.^4^ The presence of chemokines triggered monocyte arrest, and the number of arrested monocytes was recorded as measure of activity (Figure 5 A). The following three conditions were tested: OPRAH, the negative control nOPRAH (in which the N‐termini were blocked by oxime linkage at positions 1 in RANTES and PF4), and a non‐covalent dimer‐mix of RANTES and PF4. OPRAH (**17**) recruited twice as many monocytes as the non‐covalent mixed PF4 and RANTES chemokines (Figure 5 B). Furthermore, the negative control nOPRAH showed no activity, proving the importance of the free N‐terminus of OPRAH for receptor‐mediated monocyte recruitment.^12^ Peptide CKEY, which was able to disrupt non‐covalent PF4‐RANTES heterodimers and led to a decrease in atherosclerosis in a mouse model,5a was able to reduce monocyte adhesion with non‐covalent PF4‐RANTES heterodimers but not with OPRAH (Figure 5 C).

**Figure 5:**
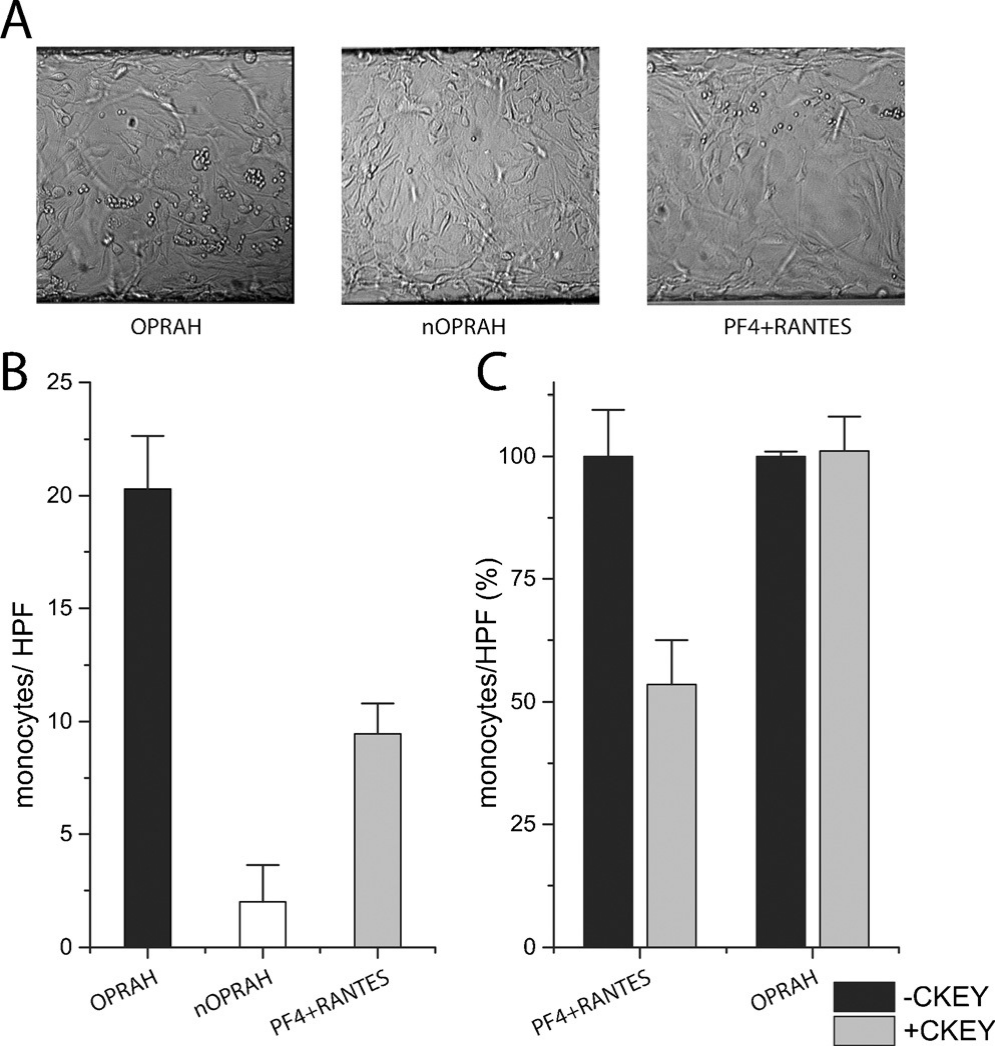
A) Microscopy images showing monocyte arrest by OPRAH, nOPRAH, and non‐covalent RANTES‐PF4 mixtures on endothelial cells. B) Monocyte arrest by OPRAH, nOPRAH (1.9 nm; *n*=6±SEM), and non‐covalent RANTES‐PF4 mixtures (3.8 nm each; *n*=3±SEM). C) Effect of CKEY on chemokine‐mediated monocyte arrest (*n*=6±SEM).

In summary, we have provided strong evidence that synergism in monocyte recruitment by the PF4‐RANTES complex proceeds in a 1:1 heterodimeric fashion. Evidence for this concept through the chemical synthesis of a covalent PF4‐RANTES heterodimer (OPRAH) additionally offers synthetic access to multiple chemokine heteromers that can unambiguously demonstrate functional enhancement through heteromerization within this novel area of chemokine biology.

## Supporting information

As a service to our authors and readers, this journal provides supporting information supplied by the authors. Such materials are peer reviewed and may be re‐organized for online delivery, but are not copy‐edited or typeset. Technical support issues arising from supporting information (other than missing files) should be addressed to the authors.

Supplementary
