# Supplementary material for: Probing Functional Heteromeric Chemokine Protein–Protein Interactions through Conformation‐Assisted Oxime Ligation
Source: Angew Chem Int Ed Engl. 2016 Oct 27;55(48):14963–6. doi: 10.1002/anie.201607036 (PMC5132021; doi:10.1002/anie.201607036)
Supplement: Supplementary file 1 — Supplementary [file ANIE-55-14963-s001.pdf]

Angewandte Chemie
Eine Zeitschrift der Gesellschaft Deutscher Chemiker

Supporting Information

Probing Functional Heteromeric Chemokine Protein–Protein Interactions through Conformation-Assisted Oxime Ligation

*Stijn M. Agten, Rory R. Koenen, Hans Ippel, Veit Eckardt, Philipp von Hundelshausen, Kevin H. Mayo, Christian Weber, and Tilman M. Hackeng**

anie_201607036_sm_miscellaneous_information.pdf

Contents

Peptide synthesis ... 2
1.1.1 Human RANTES C-term ... 2
1.1.2 Human RANTES middle (M) fragment ... 2
1.1.3 Human RANTES N-term ... 2
1.1.4 Human PF4 C-term ... 2
1.1.5 Human PF4 middle (M) fragment ... 2
1.1.6 Human PF4 N-term ... 2
Ketone or aminooxy incorporation into chemokine N-termini ... 3
Native chemical ligation of RANTES C-terminus and middle fragment (10+11) ... 3
Thiazolidine deprotection RANTES CM ligation product (12 → 14) ... 3
Native chemical ligation of RANTES CM- and N-terminus (13+14) ... 3
Oxidative folding of RANTES (15→16) ... 3
Native chemical ligation of PF4 C-terminus and middle fragment (2+3) ... 3
Thiazolidine deprotection PF4 CM ligation product (4→6) ... 4
Native chemical ligation of PF4 CM- and N-terminus (5+6) ... 4
Oxidative folding of PF4 (7→8) ... 4
HPLC analysis and purification ... 4
Mass spectrometry ... 4
UPLC mass spectrometry ... 5
Oxime ligation of PF4 and RANTES ... 5
NMR spectroscopy ... 9
Monocyte arrest assay ... 13
References ... 13

Peptide synthesis

Peptides were synthesized manually by SPPS on a 0.25 mmol scale on a MBHA or PAM resin. An *in situ* neutralization/HCTU activation for Boc chemistry was used[1]. Each synthetic cycle consisted of the Boc protective group removal by two 1-minute treatments with TFA followed by a DMF wash, addition of the pre-activated amino acid in presence of excess DIPEA, followed by another DMF wash. The Boc-protected amino acids (1.1 mmol) were activated with 1 mmol of HCTU and 3 mmol DIPEA. Coupling of Gln residues was followed by a DCM wash before and after TFA deprotection to prevent intramolecular pyrrolidone formation. After coupling of the final amino acid, the resin was dried by wash steps with DCM and DCM/MeOH.

After completion of the peptide chain, the peptides were deprotected and cleaved from the resin by treatment with anhydrous HF for 1 hour at 0° C with 4% *p*-cresol (v/v) as scavenger. Peptides were precipitated in ice-cold diethylether, dissolved in 50% acetonitrile in water + 0.1% TFA and lyophilized. The crude mixture was subjected to preparative HPLC and fractions containing the desired product were identified by ESI-MS, pooled and lyophilized.

1.1.1 Human RANTES C-term

sequence: H_2N-Cys-Ser-Asn-Pro-Ala-Val-Val-Phe-Val-Thr-Arg-Lys-Asn-Arg-Gln-Val-Cys-Ala-Asn-Pro-Glu-Lys-Lys-Trp-Val-Arg-Glu-Tyr-Ile-Asn-Ser-Leu-Glu-Met-Ser-COOH

1.1.2 Human RANTES middle (M) fragment

sequence: H_2N-Thz-Phe-Ala-Tyr-Ile-Ala-Arg-Pro-Leu-Pro-Arg-Ala-His-Ile-Lys-Glu-Tyr-Phe-Tyr-Thr-Ser-Gly-Lys-MPAL-COOH

1.1.3 Human RANTES N-term

sequence: H_2N-**Ser**-Pro-Tyr-Ser-Ser-Asp-**Thr**-Thr-Pro-Cys-MPAL-COOH

1.1.4 Human PF4 C-term

sequence: H_2N-Cys-Pro-Thr-Ala-Gln-Leu-Ile-Ala-Thr-Leu-Lys-Asn-Gly-Arg-Lys-Ile-Cys-Leu-Asp-Leu-Gln-Ala-Pro-Leu-Tyr-Lys-Lys-Ile-Ile-Lys-Lys-Leu-Leu-Glu-Ser-COOH

1.1.5 Human PF4 middle (M) fragment

sequence: H_2N-Thz-Leu-Cys-Val-Lys-Thr-Thr-Ser-Gln-Val-Arg-Pro-Arg-His-Ile-Thr-Ser-Leu-Glu-Val-Ile-Lys-Ala-Gly-Pro-His-MPAL-COOH

1.1.6 Human PF4 N-term

sequence: H_2N-**Glu**-Ala-Glu-Glu-Asp-Gly-Asp-**Leu**-Gln-MPAL-COOH

Residues in red were changed into Boc-Lys(alloc) to accommodate for ketone or aminooxy incorporations in the different constructs. Thz denotes a cysteine protected as a thiazolidine. MPAL

denotes a leucine to which a mercaptopropionic is coupled after which the sequence is continued as normal.[2]

Ketone or aminooxy incorporation into chemokine N-termini

After completion of the peptide chain, the Alloc protective group on the *N*-terminal lysine was deprotected using 2 x 30 min treatments with 0.25 eq. $Pd(PPh_3)_4$ in DCM using $PhSiH_3$ (24 eq.) as a scavenger. Subsequently, the keto-acid (4-acetyl butyric acid) or Boc (aminooxy)acetic acid (2.2 mmol) were preactivated using carbodiimide (2 mmol) (DIC) for 2 minutes after which *N*-hydroxysuccinimide (NHS) (2.0 mmol) and *N,N*-diisopropyldiamine (DIPEA) (0.6 mmol) was added, directly followed by addition to the deprotected peptide chain (0.25 mmol) and left to react for 1 hour. Finally the *N*-terminal Boc-protective group was removed by treatment with TFA as described above to result in the peptide chain modified with a ketone or aminooxy after cleavage from the resin. Alternative orthogonal protection using Fmoc was unsuccessful because of low yields obtained by deprotection with 20% piperidine (nucleophilic attack on thioester moiety) or with DBU/HOBt.

Native chemical ligation of RANTES C-terminus and middle fragment (10+11)

Both the *C*-terminus and middle fragment (M) were purified before ligation. Equimolar amounts of both parts were mixed at a concentration of 10 mg/mL in 0.1 M Tris-HCl pH 8, containing 6 M Guanidine (Gdn)-HCl, . Thiophenol was added (2% v/v). The pH was adjusted to 7 before the reaction was left to react at 37 °C for 7h with mixing every 30 minutes.

Thiazolidine deprotection RANTES CM ligation product (12 → 14)

After ligation of the C and M fragments the *N*-terminal thiazolidine was deprotected. The ligation mixture was diluted 5 times in 0.1 M Acetate pH 4 containing 6 M Guanidine (Gdn)-HCl,. MeONH2 (0.2 M) and TCEP (30 eq.) were added and the deprotection reaction was left at RT for 12h.

Native chemical ligation of RANTES CM- and N-terminus (13+14)

Both the *CM* and *N*-terminus were purified before ligation. The *N*-terminus was taken in 2 times excess, both parts were mixed at a concentration of 10 mg/mL in 0.1 M Tris-HCl pH 8, containing 6 M Guanidine (Gdn)-HCl, . Thiophenol was added (2% v/v). The pH was adjusted to 7 before the reaction was left to react at 37 °C for 1h with mixing every 5 minutes.

Oxidative folding of RANTES (15→16)

The ligated peptide was purified using HPLC, desired fractions were pooled and lyophilized. The peptide was dissolved at a concentration of 0.2 mg/mL in 0.1 M Tris-HCl pH 8 containing 3 M Gdn-HCl, . Cysteine (8 mM) and cystine (1 mM) were added and the protein was left to fold at 4 °C for 24h with continuous mixing. The folded protein was purified using HPLC, desired fractions were pooled and lyophilized.

Native chemical ligation of PF4 C-terminus and middle fragment (2+3)

Both the *C*- and Middle (M) fragments were purified before ligation. Equimolar amounts of both parts were mixed at a concentration of 10 mg/mL in 0.1 M Tris-HCl, pH 8 containing 6 M Guanidine (Gdn)-HCl, . Thiophenol and benzylmercaptan were added (1% v/v each). The pH was adjusted to 7 before the reaction was left to react at 37 °C for 6h with mixing every 30 minutes.

Thiazolidine deprotection PF4 CM ligation product (4→6)

After ligation of the C and Middle (M) fragments the *N*-terminal thiazolidine was deprotected. The ligation mixture was diluted 5 times using 0.1 M Acetate, pH 4 containing 6 M Guanidine (Gdn)-HCl, 0.2 M $MeONH_2$ and TCEP (30 eq.) The deprotection reaction was left at RT for 12h.

Native chemical ligation of PF4 CM- and N-terminus (5+6)

Both the *CM* and *N*-terminus were purified before ligation. The *N*-terminus was taken in 2 times excess, both parts were mixed at a concentration of 10 mg/mL in 0.1 M Tris-HCl, pH 8 containing 6 M Guanidine (Gdn)-HCl,. Thiophenol and benzylmercaptan were added (1% v/v each). The pH was adjusted to 7 before the reaction was left to react at 37 °C for 8h with mixing every 30 minutes.

Oxidative folding of PF4 (7→8)

The ligated peptide was purified using HPLC, desired fractions were pooled and lyophilized. The peptide was dissolved at a concentration of 0.2 mg/mL in 0.1 M Tris-HCl pH 8 containing 1 M Gdn-HCl, . Cysteine (8 mM) and cystine (1 mM) were added and the protein was left to fold at 4 °C for 24h with continuous mixing. The folded protein was purified using HPLC, desired fractions were pooled and lyophilized.

HPLC analysis and purification

Analytical HPLC was performed using a Vydac C18 HPLC column (4.6 mm x 150 mm, 1 mL/min flow rate) connected to a Varian Prostar system consisting of two Varian Prostar 215 delivery modules and a Varian Prostar 320 UV/Vis detector (λ = 214 nm). A linear gradient of 0-67 % buffer B in buffer A over 30 minutes was used, where buffer A = 0.1 v-% TFA in H_2O and buffer B = 0.1 v-% TFA, 10 v-% H_2O in CH_3CN.

Semi-preparative HPLC was performed using Vydac C18 HPLC columns (10 mm x 250 mm, 10 mL/min flow rate or 22 mm x 250 mm, 20 mL/min flow rate) connected to a Waters Deltaprep System consisting of a Waters Prep LC Controller and a Waters 2487 Dual wavelength Absorbance Detector (λ = 214 nm). Peptides were eluted using a gradient of B in A, based on an analytical HPLC run. Fractions were analyzed by Electrospray Ionization Mass Spectrometry (ESI-MS), desired fractions were pooled and lyophilized.

Mass spectrometry

UPLC ESI-MS was performed on a Waters UHPLC XEVO-G2QTOF system. Peptide masses were calculated from the experimental mass to charge (m/z) ratios of all the protonation states observed in the ESI-MS spectrum of a peptide using MaxEnt 3. Monoisotopic and average theoretical masses of compounds were calculated using Chemdraw 12.0.2.

UPLC mass spectrometry

Ligation reactions and preparative HPLC fractions were monitored on a Waters UHPLC XEVO-G2QTOF system. Sample (1-10 µL) was introduced to a PST C18 column (130A; 1.7 µm particles; column dimension 2.1x50mm, 40°C) by an FTN-autosampler, using 5% acetonitrile and 0.1% formic acid (FA) in water as purge-solvent/diluent. A water-acetonitrile gradient containing 0.1% FA (flowrate 250µL/min) was used to separate peptides and ligation products at baseline level in 14 min. Exact mass measurement was performed in resolution mode using ESI-ionisation in positive mode (sodium formate-calibrated). Both quad profile and probe settings were optimized for large peptide measurement.

Oxime ligation of PF4 and RANTES

The chemokines were dissolved in 100 mM NaOAc (pH 4.5) in equimolar amounts (200 µM) and left to react for 48h at 37 °C. Reactions were followed using UPLC-MS. Freeze catalysis was performed in identical conditions; proteins were mixed, frozen at -20 °C for 1 hour and subsequently thawed. This procedure was repeated twice .

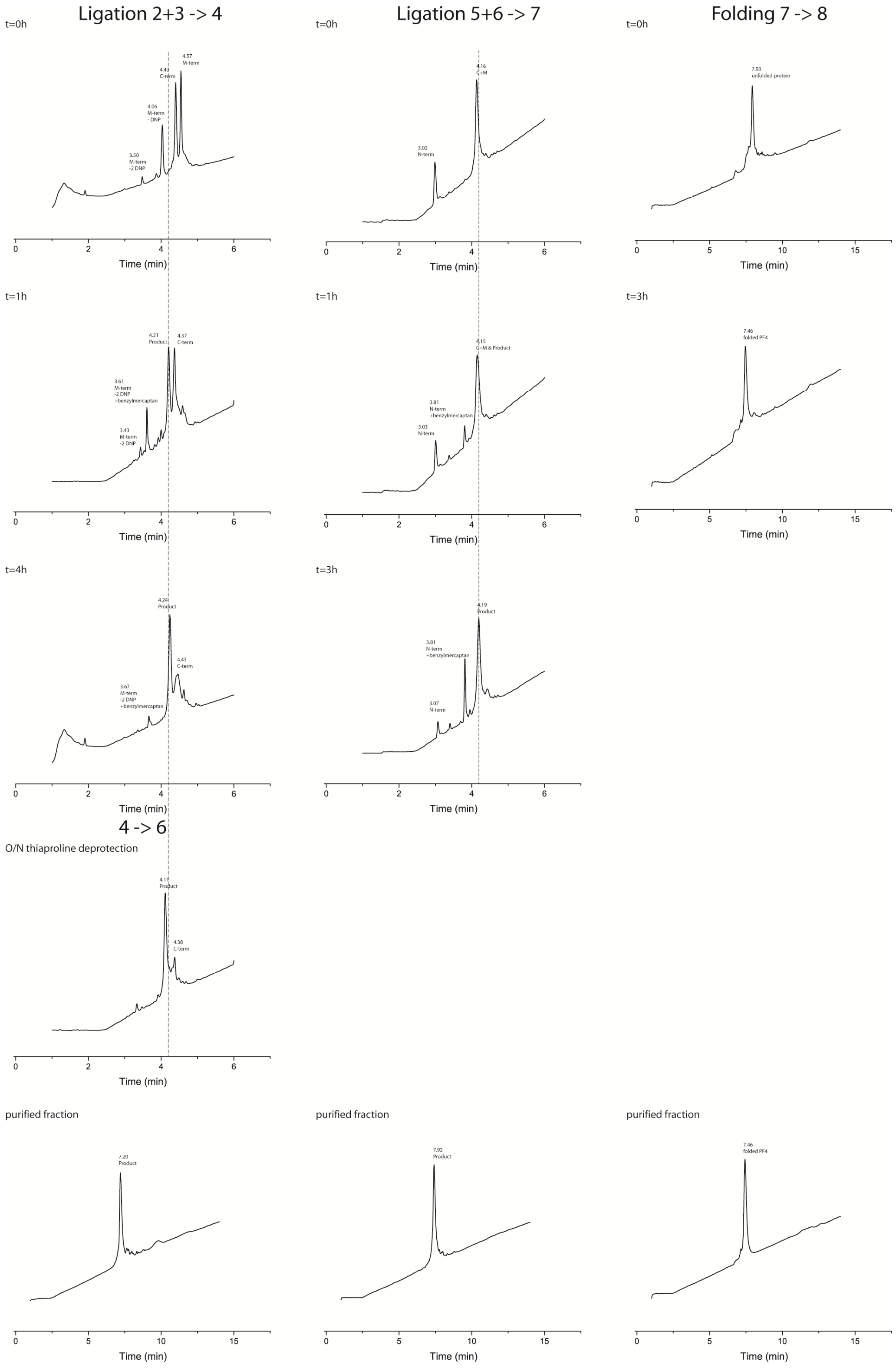

Figure SI 1, Overview of PF4 synthesis: first column shows ligation of the C-terminal with the middle fragment followed over time, with subsequent thiaproline deprotection and purification. Middle column shows NCL of the ligated product with the N-terminal fragment. The final column shows oxidative folding of PF4

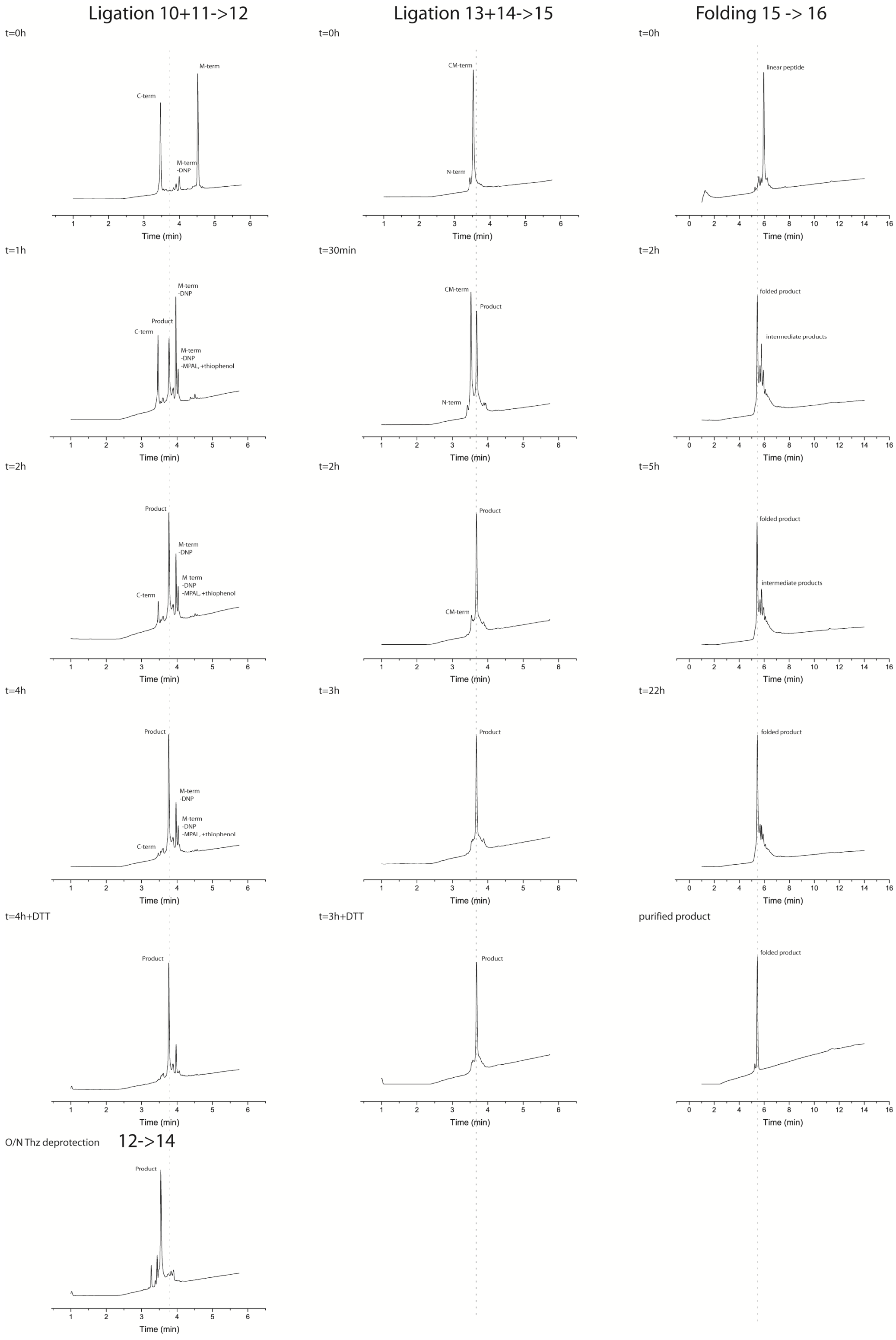

Figure SI 2, Overview of RANTES synthesis: first column shows ligation of the C-terminal with the middle fragment followed over time, with subsequent thiaproline deprotection and purification. Middle column shows NCL of the ligated product with the N-terminal fragment. The final column shows oxidative folding of RANTES

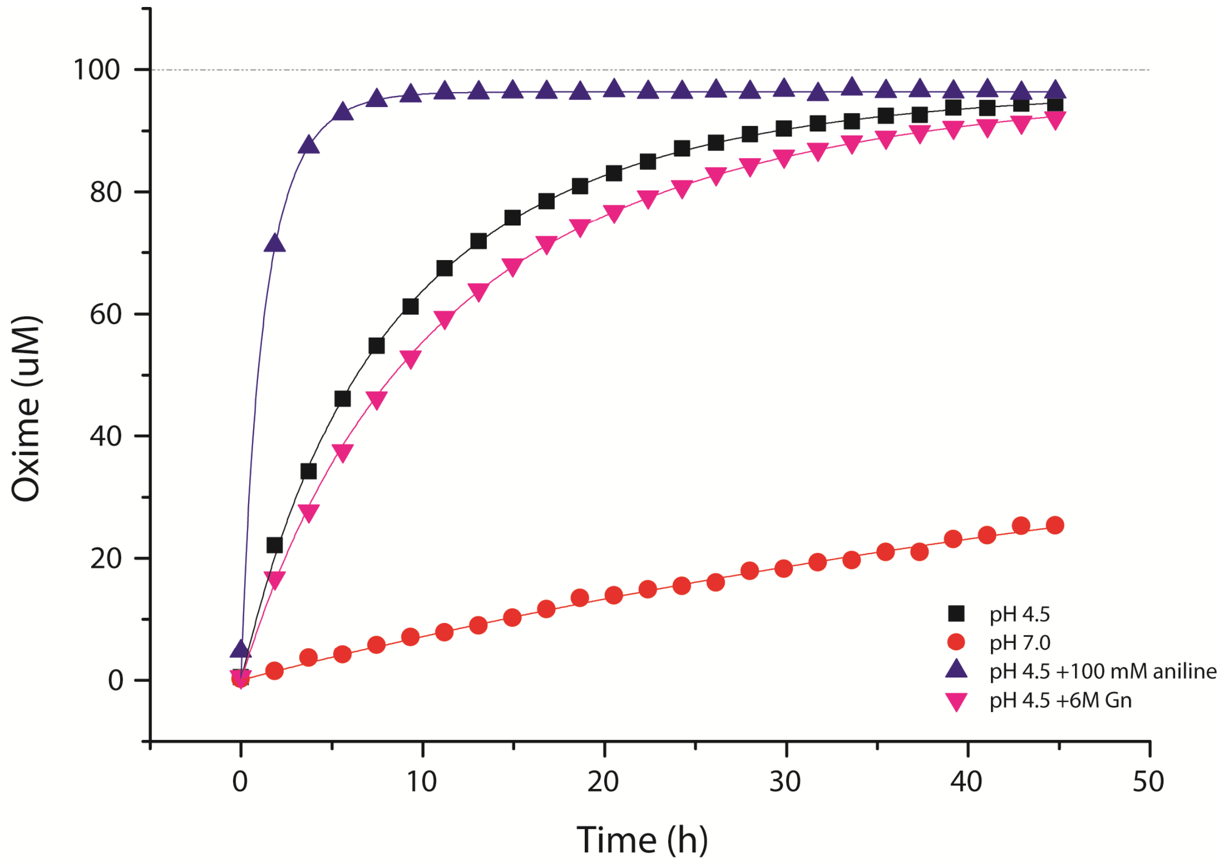

Figure SI 3, Oxime forming reaction of LYRAK(AcBu) and AO-VIPF followed over time in different conditions. All reaction were performed in 100mM NaOAc pH 4.5 or in 100mM NaPhos pH 7.0, concentrations were 100 µM for LYRAK(AcBu) and 200 µM for AO-VIPF. Conditions: ■pH 4.5 ●pH 7.0 ▲pH 4.5 +100 mM aniline ▼pH 4.5 + 6M Gdn-HCl

NMR spectroscopy

NMR samples of OPRAH, modified RANTES and modified PF4, were prepared as 0.2 mM solutions made from freeze-dried protein buffer exchanged into 25 mM deuterated NaAc-d^3 buffer (pH 3.60) containing 0.1 mM EDTA, 0.2 mM sodium azide. 5% (*v/v*) D_2O was added for deuterium lock. Initial buffer exchange steps were carried out by ultracentrifugation (in four to five steps) using pre-washed Amicon Ultra-4 ultra-centrifugaton devices with a filter cutoff of 3kDa. Final NMR solutions were prepared in Wilmad 3 mm NMR tubes (160 µl volume), containing a tiny trace of DSS for internal chemical shift calibration (0 ppm ^{1}H).

NMR spectra were recorded on a Bruker Avance III HD 700 MHz spectrometer, equipped with a cryogenically cooled TCI probe. Internal temperature was set to 37 ^oC, with the probe temperature calibrated using a thermocouple inside a NMR tube that was inserted into the probe. One dimensional proton spectra were recorded using excitation sculpting to suppress residual water. Two-dimensional DIPSI (70 ms mixing time) and 2D NOESY spectra (150 ms mixing time) were recorded using the same water suppression technique. Typically 32 scans over 600 increments were collected per spectrum. In addition, natural abundance heteronuclear ^{13}C-^{1}H HSQC spectra were acquired for better comparison to known assignments of free RANTES and PF4.

The oxime reaction between RANTES and RANTES to form OPRAH was followed in real-time by 1D NMR inside the NMR tube at 50 ^oC. The reaction proceeds relatively slowly, so for that reason the reaction was also studied by freezing the NMR sample at -20 ^oC for one hour in the freezer, and slowly warm up the sample back to room temperature.

Spectra processing was performed by Bruker Topspin3.2 and Sparky 3.114 software.

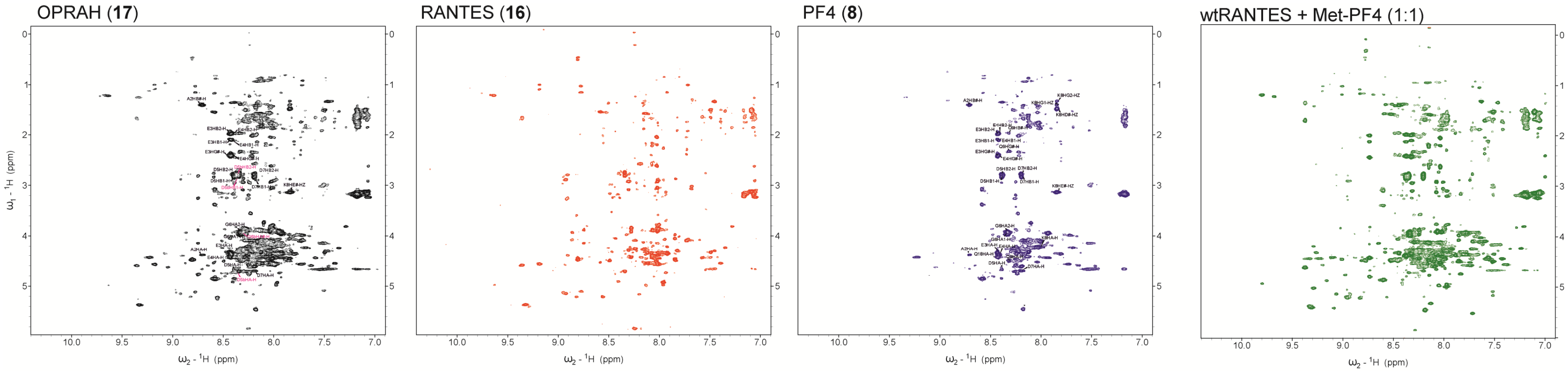

Figure SI 4, Comparison between the DIPSI spectra (70 msec mixing time) of 0.2 mM OPRAH (17), RANTES(16) PF(8) and a 1:1 complex of wtRANTES + Met-PF4 measured under the same conditions (700 MHz, 37 °C in 25 mM NaAc-d3 buffer (pH 3.60), 0.1 mM EDTA, and 0.2 mM sodium azide). Assignments for native tetrameric PF4 are incomplete, mostly due to severe dynamic chemical shift broadening of proton resonances, N-terminal amide resonances of PF4(2-7) are however sharp and sequentially assigned from NOESY spectra. Analysis show that the DIPSI spectrum of OPRAH (black) mostly corresponds to a superposition of the spectra of two separately folded domains. Signals for important residues connected just around the linker region are however missing in OPRAH, most likely due to dynamic repositioning between the two domains on an intermediate millisecond time scale.

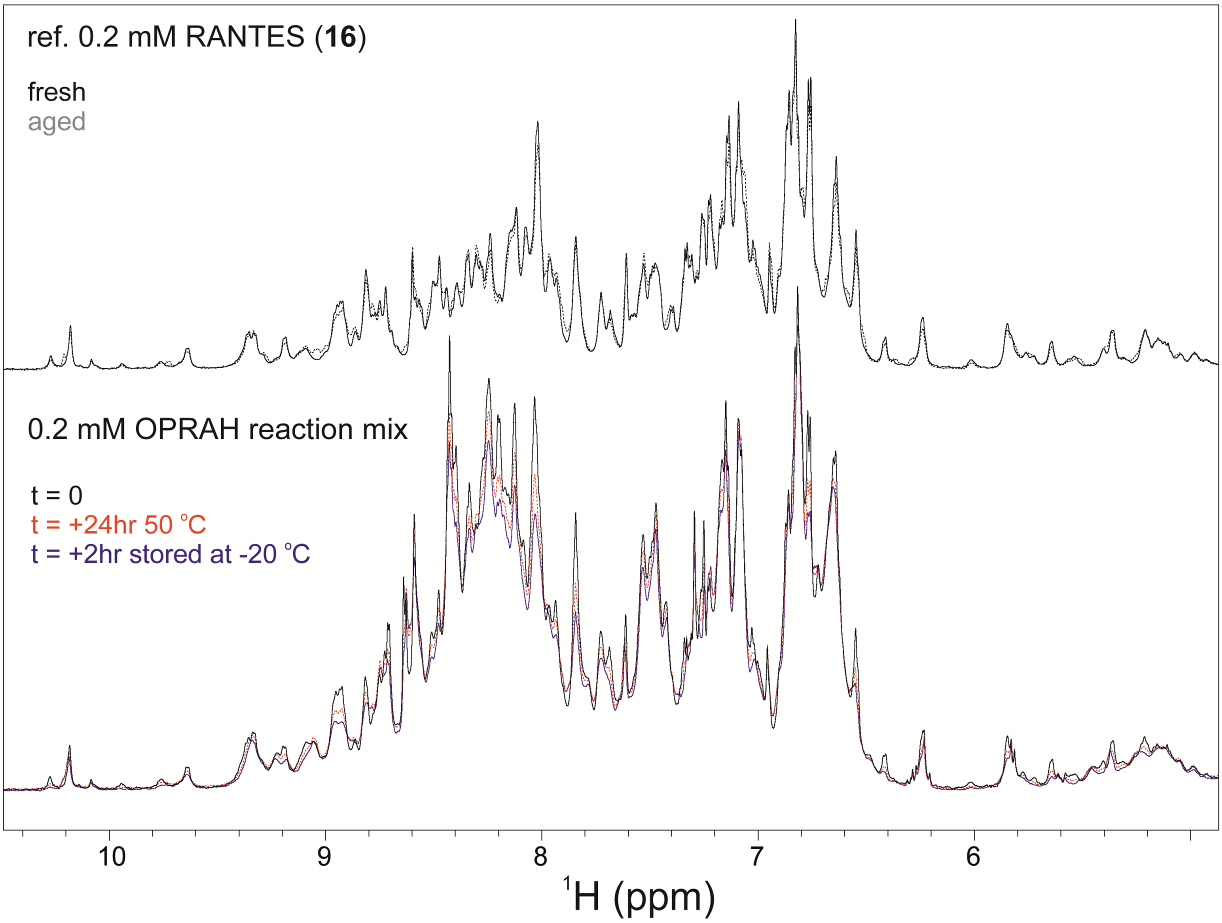

Figure SI 5, Bottom spectrum: Comparison of ^{1}H NMR 700 MHz spectra (amide region) monitoring the oxime reaction between 0.2 mM RANTES (16) and 0.2 mM PF4 (8) yielding OPRAH. Reaction times were +24 hour at 50 °C (red), and +2 hr at -20 °C, followed by thawing and a warm up of the frozen NMR sample (blue). Top spectrum: reference spectrum of free 0.2 mM RANTES (16) taken at time zero (black) and +24 hours at 50 °C (broken line). Changes have been contributed to side reactions of the aminooxy group in free RANTES (16) and gradually appear in both the spectrum of RANTES and OPRAH after keeping the sample for several hours at 50 °C (broken line).

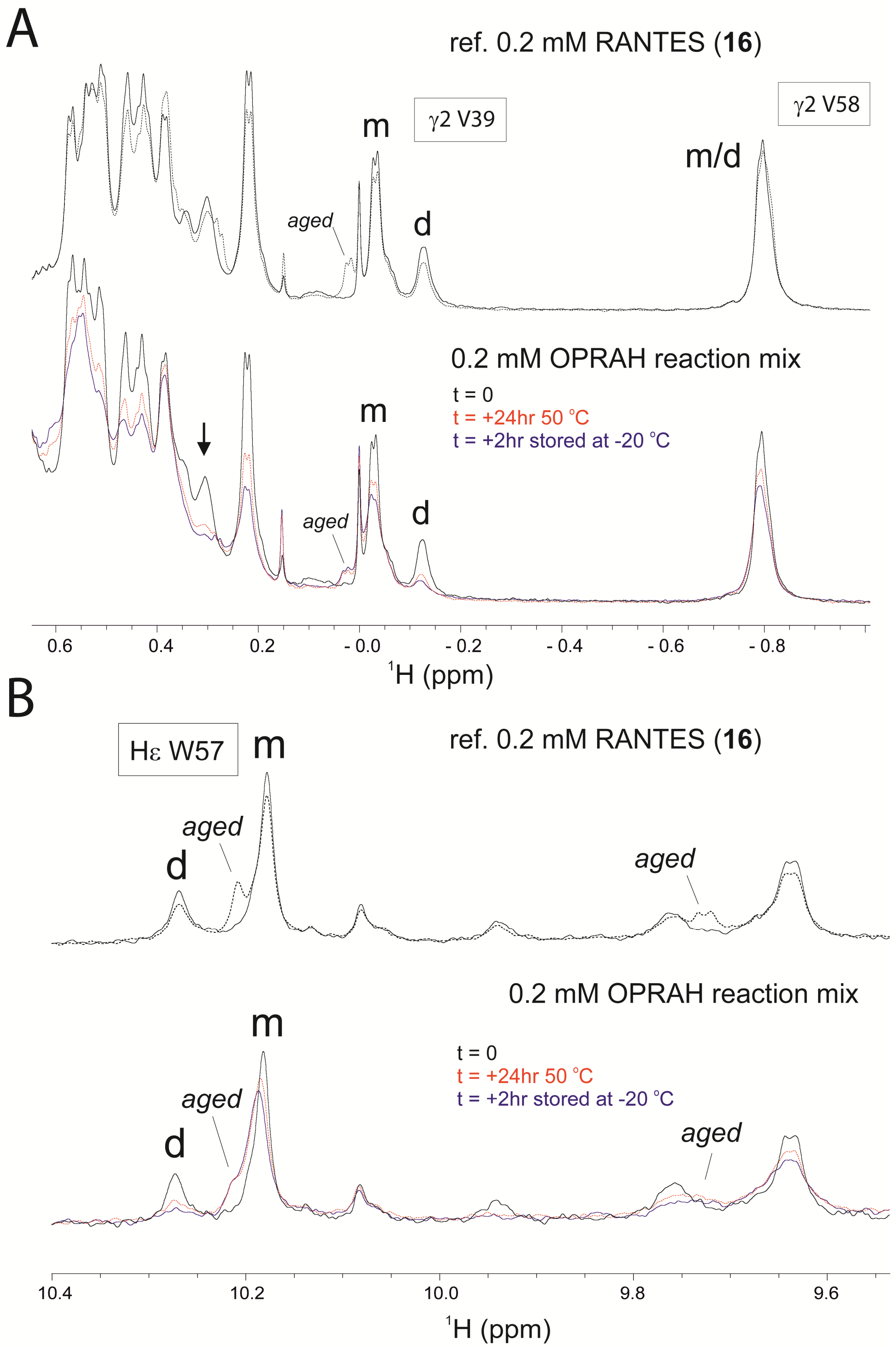
A
ref. 0.2 mM RANTES (16)
γ2 V39
γ2 V58
m
m/d
aged
d
0.2 mM OPRAH reaction mix
t = 0
t = +24hr 50 °C
t = +2hr stored at -20 °C
m
aged
d
0.6
0.4
0.2
- 0.0
- 0.2
- 0.4
- 0.6
- 0.8
¹H (ppm)
B
Hε W57
m
ref. 0.2 mM RANTES (16)
aged
d
aged
0.2 mM OPRAH reaction mix
m
t = 0
t = +24hr 50 °C
t = +2hr stored at -20 °C
aged
d
aged
10.4
10.2
10.0
9.8
9.6
¹H (ppm)

Figure SI 6, A: Top spectrum: methyl groups in the reference RANTES (16) protein. Two different conformational states of the RANTES domain are present: m = monomeric; d = dimeric (top) in both RANTES and OPRAH. Peaks denoted "aged" have been contributed to side reactions of the aminooxy group of free RANTES (16) and gradually appear in both the spectrum of RANTES and OPRAH after keeping the sample for several hours at 50 °C (broken line). Most prominent change of conformational state in RANTES (16) is the diminished dimer population compared to native RANTES, due to steric hindrance of the introduced oxime bridge involving lysine sidechain at RANTES position T7K. Bottom spectrum: Comparison of ^{1}H NMR 700 MHz spectra (methyl region) monitoring the oxime reaction between 0.2 mM RANTES (16) and 0.2 mM PF4 (8) yielding OPRAH. Reaction times were +24 hour at 50 °C (red), and +2 hr at -20 °C, followed by thawing and a warm up of the frozen NMR sample (blue). After freezing the reaction NMR mixture for an additional +2 hours at -20 °C, mass spectra indicate near-complete conversion to OPRAH, and the population of homodimer RANTES states decrease even more compared to free RANTES (16), in agreement with competitive formation of heterodimers formed between RANTES and PF4 in OPRAH. **B:** Enlarged section of Figure SI5 showing the comparison of ^{1}H NMR 700 MHz spectra (Trp sidechain amino region) monitoring the oxime reaction between 0.2 mM RANTES (16) and 0.2 mM PF4 (8) yielding OPRAH. Reaction times were +24 hour at 50 °C (red), and +2 hr at -20 °C, followed by thawing and a warm up of the frozen NMR sample (blue). Top spectrum: Peaks of RANTES Trp57 Hε in the reference RANTES (16) protein. Two different conformational states of the RANTES domain are present: m = monomeric; d = dimeric (top) in both RANTES and OPRAH. Peaks denoted "aged" have been contributed to side reactions of the aminooxy group of free RANTES (16) and gradually appear in both the spectrum of RANTES and OPRAH after keeping the sample for several hours at 50 °C (broken line). Bottom spectrum: Comparison of ^{1}H NMR 700 MHz spectra (aromatic region) monitoring the oxime reaction between 0.2 mM RANTES (16) and 0.2 mM PF4 (8) yielding OPRAH. Reaction times were +24 hour at 50 °C (red), and +2 hr at -20 °C, followed by thawing and a warm up of the frozen NMR sample (blue)

Monocyte arrest assay

$CD14^{+}$ human monocytes were isolated from peripheral blood mononuclear cells (PBMC), which were separated from human whole blood (see above), and purified by negative selection using the "Monocyte Isolation Kit II" according to the manufacturers protocol (Miltenyi Biotec GmbH, Bergisch Gladbach, Germany). Human aortic endothelial cells (HAoEC) were cultured and activated with IL-1ß for 4 hours prior to incubation for 30 minutes with chemokines (CXCL4+CCL5 3.8 nM) or synthetic obligate chemokine (1.9 nM) heterodimers and peptide interfering with heterodimerization inhibitor (CKEY). Monocytes (0.7×10^{6}/ml) were applied onto the activated endothelium shear stress (1.5 dyn/cm^{2}) using a syringe pump (WPI, Berlin, Germany) in a laminar flow chamber for 2 min and flushed with assay medium thereafter. Cells were visualized and recorded using a phase contrast microscope (Olympus IX 50, Shinjuku, Japan) connected to a CMOS camera (ids, Obersulm, Germany). For quantification, tightly adherent leukocytes were manually counted in 10 fields of view per dish. Experiments were performed n=3 for (CXCL4+CCL5) or n=6 for OPRAH and nOPRAH. Presented data were subtracted with monocyte adherence in absence of chemokines. Data are presented as mean +SEM.

References

[1] M. Schnolzer, P. Alewood, A. Jones, D. Alewood, S. B. Kent, *Int J Pept Protein Res* **1992**, *40*, 180-193.
[2] T. M. Hackeng, J. H. Griffin, P. E. Dawson, *Proc Natl Acad Sci U S A* **1999**, *96*, 10068-10073.
